# Sex differences in ferroptosis-related vulnerability to autism-like deficits in the adolescent medial prefrontal cortex following embryonic valproic acid exposure

**DOI:** 10.1186/s13293-026-00982-x

**Published:** 2026-09-15

**Authors:** Shatong Zhao, Jing Wang, Yi Pan, Ying Yang, Yifei Yin, Wu Li, Jiangshan Li, Xiang Feng

**Affiliations:** 1 School of Acupuncture-Moxibustion, Tuina and Rehabilitation, Hunan University of Chinese Medicine, Changsha, Hunan 410208 China; 2 School of Medicine, Hunan University of Chinese Medicine, Changsha, Hunan 410208 China

**Keywords:** Autism spectrum disorder, Valproic acid, Sex differences, Ferroptosis, Medial prefrontal cortex

## Abstract

**Background:**

Prenatal valproic acid (VPA) exposure is an important environmental risk factor for autism spectrum disorder (ASD), but the mechanisms underlying VPA-induced ASD-like phenotypes remain unclear. Emerging evidence implicates neuronal ferroptosis and neurodevelopmental disturbances, yet whether these alterations vary across developmental stages and between sexes is unknown.

**Methods:**

Adolescent male and female C57BL/6 mice exposed to VPA at embryonic day 12.5 (E12.5) or postnatal day 14 (PND14) were used to examine behavioral and neurobiological effects of VPA exposure across developmental stages and between sexes. Sociability and anxiety-like behaviors were assessed using the three-chamber social interaction test (3-CT) and open field test (OFT), respectively. Histological alterations in the medial prefrontal cortex (mPFC), hippocampus, and striatum were examined using hematoxylin-eosin (HE) and Nissl staining. Ferroptosis-related changes were evaluated using biochemical assays, immunofluorescence, transmission electron microscopy and Western blotting. In male mice, the effects of prenatal and early postnatal VPA exposure were compared to assess whether similar behavioral and ferroptosis-related alterations were observed across developmental windows.

**Results:**

Both male and female adolescent offspring exposed to VPA exhibited social deficits and anxiety-like behaviors, with a more pronounced behavioral deficits in males, particularly in social preference and sociability. Histological analysis revealed variable degrees of neuronal abnormalities in the mPFC and hippocampus of VPA-exposed offspring, with the most prominent alterations observed in the mPFC. These histological changes were observed in both male and female offspring. Ferroptosis-related molecular changes were most evident in the mPFC and were accompanied by mitochondrial abnormalities and vacuolization. These alterations exhibited a distinct pattern of sex differences, with more pronounced changes observed in males than in females. Additionally, in male offspring, prenatal and early postnatal VPA exposure were associated with similar patterns of behavioral abnormalities and ferroptosis-related changes in the mPFC.

**Conclusion:**

This study suggests that prenatal VPA exposure induces ferroptosis-related abnormalities which could be linked to ASD-like behavioral and functional impairments, with more pronounced changes observed in male offspring. Among the brain regions examined, the mPFC showed the most prominent neurohistological and ferroptosis-related alterations. In male mice, prenatal and early postnatal VPA exposure were associated with similar patterns of ferroptosis-related molecular alterations, suggesting that ferroptosis-assiociated alterations may represent a shared molecular feature of VPA-induced neurodevelopmental abnormalities across different developmental windows.

**Graphical Abstract:**

**Figure:**
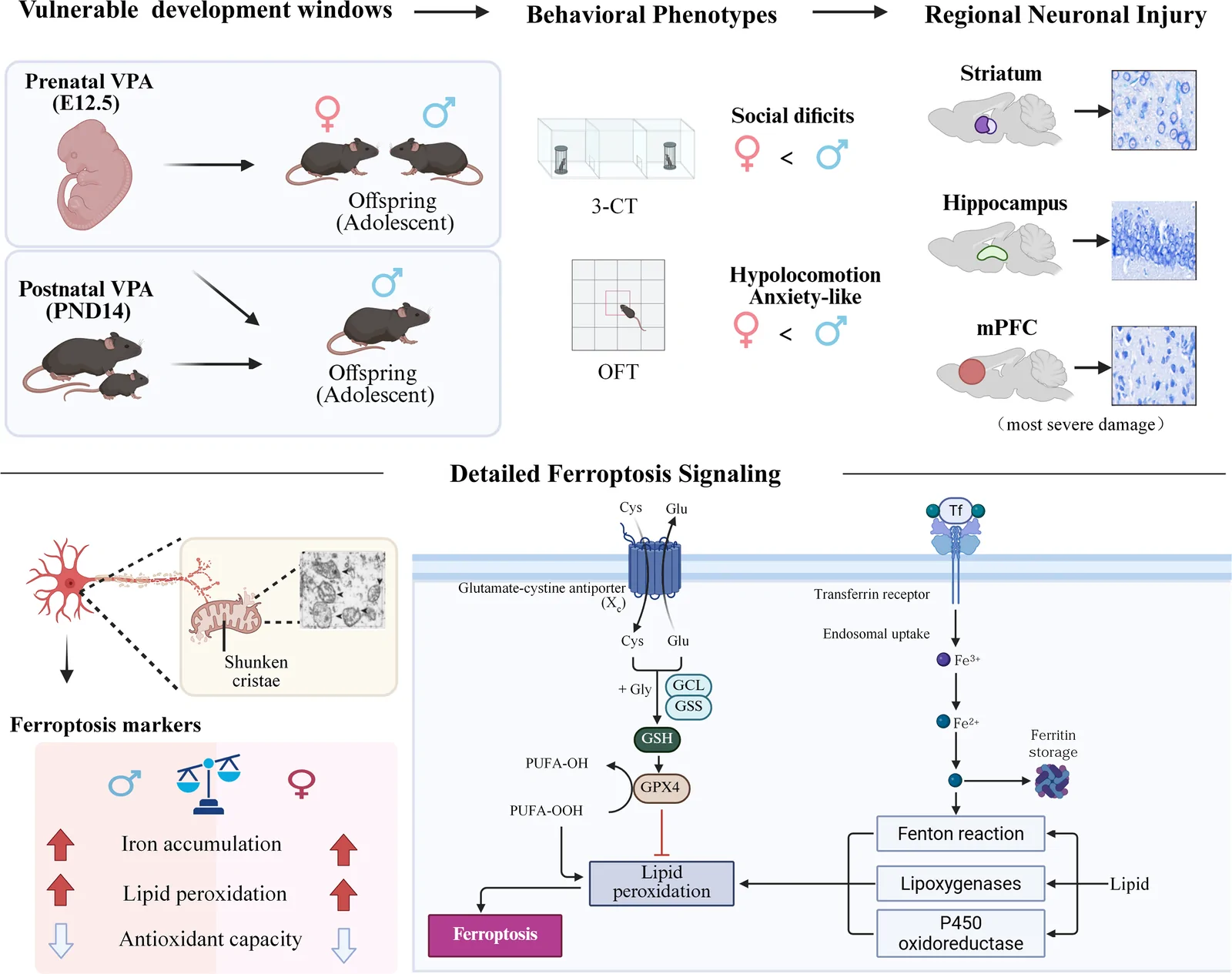

**Supplementary Information:**

The online version contains supplementary material available at https://doi.org/10.1186/s13293-026-00982-x.

## Plain language summary

Autism is diagnosed more often in boys than in girls, but the biological reasons for this sex difference remain unclear. Valproic acid (VPA) is used to treat epilepsy and certain mood disorders, although prenatal exposure has been associated with an increased risk of autism-related developmental impairments. In our study, we explored whether VPA affects the developing brain differently in male and female mice. We used a well-established mouse model in which animals were exposed to VPA before birth. When the mice reached adolescence, we examined their social behavior, activity, and anxiety-like behavior. We also looked at several brain areas, including the medial prefrontal cortex (mPFC), which has been implicated in social behavior, emotional processing, and decision-making. We then compared male mice exposed to VPA before birth with male mice exposed during early life. Our findings showed that VPA exposure reduced social behavior and increased anxiety-like behavior in both male and female mice, with sex-related differences in the magnitude of these behavioral alterations. The clearest brain changes were found in the mPFC, where neuronal alterations were observed in both male and female mice. We also found signs of iron-related cell stress, known as ferroptosis, particularly in the mPFC. Similar patterns of behavioral and ferroptosis-related changes were observed in male mice following exposure before birth or during early life. Overall, our findings suggest that ferroptosis-related changes in the mPFC are associated with the more pronounced autism-like behavioral changes observed in male mice following VPA exposure.

**Supplementary Information:**

The online version contains supplementary material available at https://doi.org/10.1186/s13293-026-00982-x.

## Highlights

- Prenatal VPA exposure induced social deficits and anxiety-like behaviors in adolescent mice, with significant differences observed between sexes.
- Prenatal VPA exposure was associated with region-specific neuronal alterations, with the most prominent changes observed in the mPFC.
- Prenatal VPA exposure was associated with ferroptosis-related alterations across the mPFC, hippocampus, and striatum, with sex-related differences most evident in the mPFC.
- Prenatal and early postnatal VPA exposure were associated with similar patterns of behavioral deficits and mPFC ferroptosis-related alterations in male mice.
- These findings suggest that ferroptosis-related alterations in the mPFC may represent a shared molecular feature associated with VPA-induced neurodevelopmental abnormalities across the developmental exposure windows.

**Supplementary Information:**

The online version contains supplementary material available at https://doi.org/10.1186/s13293-026-00982-x.

## Introduction

Autism spectrum disorder (ASD) is a complex neurodevelopmental disorder characterized by persistent deficits in social communication and interaction, alongside restricted and repetitive behavioral patterns, interests, or activities [1–3]. The etiology of this disorder is thought to arise from the intricate interplay of genetic susceptibility, environmental exposure, and developmental factors [4–6]. A significant epidemiological characteristic is the sex bias in diagnosis, which reveals a markedly higher rate of diagnosis among men compared to women [7–10]. However, existing evidence indicates that a higher diagnosis rate of ASD in men than in women does not necessarily equate to a gender difference in the actual prevalence rate [7, 8, 11, 12]. Female individuals may mask their autistic characteristics through social disguises and compensatory strategies, thereby reducing the clinical recognition rate and diagnosis rate [13–15]. These factors may partially explain the higher diagnosis rate of ASD in men, but the underlying biological and pathological basis still needs to be further clarified.

Previous studies have shown that susceptibility to ferroptosis may exhibit sex dimorphism [16–18]. In the sodium valproic acid (VPA)-induced ASD model, ferroptosis was observed in dopaminergic neurons of the ventral tegmental area (VTA), implicating these neurons in the pathological processes associated with ASD [19]. Ferroptosis represents an iron-dependent regulatory form of cell death that is distinct from apoptosis [20–22]. Its primary characteristic is the accumulation of iron ions within cells, which leads to the excessive generation of membrane lipid peroxides, ultimately resulting in cellular damage and death. Neurons may exhibit increased susceptibility to ferroptosis-related damage because their membrane lipids are abundant in polyunsaturated fatty acids, they possess elevated levels of oxidative metabolism, and they rely heavily on the antioxidant defense system [23–26]. Significant sex differences exist in the effects of prenatal VPA exposure on male and female offspring. For example, in Wistar rats, female offspring demonstrated a lower susceptibility to impairments in social communication, emotional response, and cognitive performance resulting from prenatal VPA exposure, whereas male offspring displayed more pronounced deficits in social skills [27]. A separate study involving mice also indicated that prenatal VPA exposure could lead to a reduction in social skills among male offspring, while female offspring did not exhibit similar deficiencies [28]. Consequently, it is imperative to further investigate whether neuronal ferroptosis exhibits sexual dimorphism in VPA-induced ASD model mice and whether this disparity may elucidate a potential biological mechanism underlying the male bias associated with ASD.

Recent research indicates that following intraperitoneal administration of sodium VPA to mice on PND14, ferroptosis becomes evident in the hippocampal area on P15 [29]. In a separate investigation, exposure to prenatal VPA at E12.5 led to alterations associated with ferroptosis in the VTA of mice at P30 [19]. These findings imply that VPA-induced ferroptosis may not be restricted to particular developmental stages or brain regions but represents a dynamic process persisting throughout developmental phases and contributing to the pathological advancement of ASD. Since the introduction of the prenatal E12.5 high-dose VPA exposure model in 1996 [30], this model has been extensively employed to induce autism-like behaviors in rodents. The E12.5 period corresponds to the early stages of pregnancy following the closure of the human neural tube, representing a crucial window for nervous system development [31]. In contrast, the postnatal VPA model typically targets the early post-birth stage in rodents, specifically from P0 to P14. This timeframe aligns with the brain development phase from the perinatal period to early infancy in humans, during which neural development is highly active and susceptible to external influences [29, 32–35]. Notably, although both prenatal and postnatal VPA exposure can induce autism-like behaviors accompanied by ferroptosis-related alterations, it remains unclear whether VPA-induced ferroptosis differs across developmental windows, particularly in terms of its molecular regulatory pathways and severity.

This study aims to establish a prenatal model of ASD induced by VPA through intraperitoneal injection into pregnant mice on the E12.5. First, by comparing behavioral manifestations, alterations in brain tissue morphology, and molecular indicators associated with ferroptosis between male and female offspring, this research investigates the potential involvement of ferroptosis in the sex bias observed in ASD. Second, we further examined ferroptosis-related molecular alterations, together with behavioral and brain morphological changes, in adolescent mice following VPA exposure at two distinct developmental windows, prenatal E12.5 and early postnatal PND14. This comparison aimed to determine whether ferroptosis-related alterations differ between developmental windows or represent a convergent pathological response associated with VPA exposure across these stages.

## Materials and methods

### Animals

Specific pathogen-free C57BL/6 mice were obtained from the Laboratory Animal Center of Hunan University of Chinese Medicine. All animals were allowed to acclimatize for one week before experimental procedures. In the animal department, each cage typically housed five mice. Animals were provided ad libitum access to a maintenance diet for mice (Product no.1025, Beijing, China), providing 3.42 kcal/g and containing 19.2% crude protein, 4.6% crude fat, and 4.0% crude fiber. The full composition is provided in Supplementary Table S1. Food and water were available ad libitum. Animals were maintained under a non-reversed 12-h light/dark cycle, with lights on at 07:00 and lights off at 19:00. Room temperature was maintained at 23℃-25℃ and relative humidity at 40%-60%. After grouping, mice from different treatment conditions were housed in separate group cages, with no more than five mice per cage, to prevent cross-group mixing and potential environmental contamination. To minimize litter-related bias, one male and one female offspring were randomly selected from each litter for subsequent grouping and analysis. A total of 64 offspring mice were included in this study. All procedures, including diet and experimental protocols, were conducted in strict adherence to the guidelines of the Institutional Animal Care and Use Committee of Hunan University of Chinese medicine (Approval no.LL2023042603) and in compliance with international standards for the ethical treatment of experimental animals.

### VPA administration and ASD model

Adult male and female mice aged 6–8 weeks were used for breeding. All breeding mice were allowed to acclimate for one week under standard housing conditions before mating. Female and male mice were then co-housed overnight at a female-to-male ratio of 2:1. Each female mouse was used for only one pregnancy to minimize variability associated with repeated breeding and maternal physiological changes. The morning on which a vaginal plug was detected was designated as E0.5. On E12.5, pregnant dams were randomly assigned to either the VPA group or the saline control group. Pregnant dams in the VPA group received a single intraperitoneal injection of VPA at 500 mg/kg dissolved in sterile saline [36, 37], whereas dams in the control group received an equal volume of sterile saline at the same time point. Offspring prenatally exposed to VPA were used to model ASD-like phenotypes, while offspring from saline-treated dams served as controls. In a separate cohort, a postnatal VPA exposure model was established. Pups in the postnatal VPA group received a single intraperitoneal injection of VPA at 400 mg/kg dissolved in sterile saline on PND14 [29], whereas pups in the corresponding control group received an equal volume of sterile saline. Pups were monitored for developmental parameters, including body weight, naso-anal length (hereafter referred to as body length) and tail length. Body length and tail length were measured using a flexible measuring tape graduated in 1-mm increments. Mice were gently restrained in a standardized natural posture without stretching the body or tail. All measurements were performed at approximately the same time of day by an experimenter blinded to group allocation to minimize potential measurement bias and circadian variability. After weaning, mice were group-housed according to sex and treatment condition.

### Behavioral studies

Behavioral tests were performed from PND22 to PND23, with all testing conducted between 12:00 and 18:00 during the light phase of the regular light/dark cycle. Animals from different treatment groups and sexes were tested in a randomized and balanced order across the testing window to minimize potential systematic effects of time of day and testing order. Specifically, the three-chamber social interaction test (3-CT) was conducted on PND22, followed by the open field test (OFT) on PND23, and the same testing sequence was used for all animals. Behavioral testing was conducted in a quiet room under controlled illumination, with light intensity maintained at approximately 150 lx. Temperature and humidity in the testing room were maintained at 23.5 ± 2.5 °C and 45 ± 5%, respectively. Before each test, mice were allowed to acclimate to the testing room for 30 min to reduce stress associated with environmental transfer. After each trial, the apparatus was thoroughly cleaned with 70% ethanol and allowed to dry completely before the next animal was tested, in order to minimize residual olfactory cues. Following completion of the behavioral tests, mice were anesthetized and perfused on PND24. The brains were rapidly removed and placed on ice, and the olfactory bulbs and lower brainstem were carefully excised. The remaining brain tissue was immediately weighed using an analytical balance to determine wet brain weight. Brain tissues were subsequently dissected and collected for histological and molecular biological analyses according to the requirements of each experiment.

### Three chamber social interaction test (3-CT)

To assess sociability and social novelty preference in mice [38], the three-chamber sociability and social novelty test was performed using an opaque rectangular apparatus divided into three interconnected chambers of equal size by two transparent partitions with openings allowing free access between chambers. Each chamber measured 20 cm W × 40 cm L × 40 cm H. The test consisted of three phases. During the habituation phase, the test mouse was allowed to freely explore all three chambers for 10 min. In the sociability phase, a circular wire cage containing an unfamiliar age-, sex-, and strain-matched mouse, designated Stranger 1 (S1), was placed in one lateral chamber, whereas an identical empty wire cage (E) was placed in the opposite lateral chamber. The positions of S1 and the empty cage were counterbalanced across animals to avoid side preference. The test mouse was then allowed to freely explore all three chambers for 5 min. In the social novelty phase, a second unfamiliar age-, sex-, and strain-matched mouse, designated Stranger 2 (S2), was placed in the previously empty wire cage, while S1 remained in its original cage. The test mouse was allowed to explore the apparatus for another 5 min. Stranger mice were habituated to the wire cages before testing to reduce stress-related movement during the experiment. Time spent in the 2-cm interaction zone surrounding each wire cage and the cumulative duration of direct sniffing toward each wire cage were recorded and analyzed using Tracking Master V5. The apparatus and wire cages were cleaned with 70% ethanol and allowed to dry completely between trials to minimize residual olfactory cues. The sociability index and social novelty index were calculated as follows:

$$\begin{aligned}&\rm Social\:novelty\:index \cr&=\:\frac{\begin{aligned}&\mathrm{T}\mathrm{i}\mathrm{m}\mathrm{e}\:\mathrm{s}\mathrm{p}\mathrm{e}\mathrm{n}\mathrm{t}\:\mathrm{w}\mathrm{i}\mathrm{t}\mathrm{h}\:\mathrm{S}\mathrm{t}\mathrm{r}\mathrm{a}\mathrm{n}\mathrm{g}\mathrm{e}\mathrm{r}\:2\:\cr&-\:\mathrm{T}\mathrm{i}\mathrm{m}\mathrm{e}\:\mathrm{s}\mathrm{p}\mathrm{e}\mathrm{n}\mathrm{t}\:\mathrm{w}\mathrm{i}\mathrm{t}\mathrm{h}\:\mathrm{S}\mathrm{t}\mathrm{r}\mathrm{a}\mathrm{n}\mathrm{g}\mathrm{e}\mathrm{r}\:1\end{aligned}}{\mathrm{T}\mathrm{o}\mathrm{t}\mathrm{a}\mathrm{l}\:\mathrm{t}\mathrm{i}\mathrm{m}\mathrm{e}\:\mathrm{s}\mathrm{p}\mathrm{e}\mathrm{n}\mathrm{t}\:\mathrm{w}\mathrm{i}\mathrm{t}\mathrm{h}\:\mathrm{b}\mathrm{o}\mathrm{t}\mathrm{h}}\end{aligned}$$

$$\begin{aligned}&\rm Sociability\:index\cr&=\:\frac{\begin{aligned}&\mathrm{T}\mathrm{i}\mathrm{m}\mathrm{e}\:\mathrm{s}\mathrm{p}\mathrm{e}\mathrm{n}\mathrm{t}\:\mathrm{w}\mathrm{i}\mathrm{t}\mathrm{h}\:\mathrm{S}\mathrm{t}\mathrm{r}\mathrm{a}\mathrm{n}\mathrm{g}\mathrm{e}\mathrm{r}\:1\:\cr&-\:\mathrm{T}\mathrm{i}\mathrm{m}\mathrm{e}\:\mathrm{s}\mathrm{p}\mathrm{e}\mathrm{n}\mathrm{t}\:\mathrm{w}\mathrm{i}\mathrm{t}\mathrm{h}\:\mathrm{e}\mathrm{m}\mathrm{p}\mathrm{t}\mathrm{y}\:\mathrm{c}\mathrm{a}\mathrm{g}\mathrm{e}\end{aligned}}{\mathrm{T}\mathrm{o}\mathrm{t}\mathrm{a}\mathrm{l}\:\mathrm{t}\mathrm{i}\mathrm{m}\mathrm{e}\:\mathrm{s}\mathrm{p}\mathrm{e}\mathrm{n}\mathrm{t}\:\mathrm{w}\mathrm{i}\mathrm{t}\mathrm{h}\:\mathrm{b}\mathrm{o}\mathrm{t}\mathrm{h}}\end{aligned}$$

### Open field test (OFT)

The OFT was conducted in an open-field arena measuring 50 cm W × 50 cm L × 40 cm H [39]. The arena was divided into a central zone and a peripheral zone. The floor was virtually divided into 16 equal squares; the central four squares were defined as the central zone, and the remaining 12 squares were defined as the peripheral zone. At the beginning of the test, each mouse was gently placed in the center of the arena in a consistent manner and allowed to explore freely for 5 min. The total distance traveled and the time spent in each zone were recorded and analyzed using Tracking Master V5. The arena was cleaned with 70% ethanol and allowed to dry completely between trials. Locomotor activity was assessed based on the total distance traveled, whereas anxiety-like behavior was evaluated based on the time spent in the central zone.

### Western blotting

Mice were deeply anesthetized with pentobarbital sodium (70 mg/kg, intraperitoneally) and transcardially perfused with ice-cold phosphate-buffered saline (PBS) on PND24 to remove blood contamination before tissue collection. Tissue collection was performed at the same time of day for all animals to minimize circadian-related variability. All tissues used for molecular analyses were collected at PND24. Protein levels of GPX4, ACSL4, FTH1, and xCT/SLC7A11 were assessed in the mPFC, striatum, and hippocampus. The mPFC, striatum, and hippocampus were rapidly dissected on ice, immediately frozen, and stored at -80 °C until protein extraction. Protein lysates were prepared using RIPA lysis buffer (Cat# CW2333S; CWBIO, China) supplemented with 1% protease inhibitor cocktail (Cat# A1320914-1 ml; Ambeed, USA) and 1% phosphatase inhibitor cocktail (Cat# A2679231; Ambeed, USA). Tissues were homogenized in lysis buffer, incubated on ice for 30 min, and centrifuged at 12,000 × g for 15 min at 4 °C. The supernatants were collected, and protein concentrations were measured using a bicinchoninic acid (BCA) kit (Cat# E-BC-K318-M; Elabscience, China). Protein samples were mixed with loading buffer and denatured at 100 °C for 10 min. A total of 10 µg of protein was loaded onto 12% SDS-PAGE gels for electrophoretic separation and then transferred to 0.45 μm polyvinylidene fluoride (PVDF) membranes (Merck Millipore, Burlington, MA, USA). After transfer, the membranes were blocked with 5% non-fat milk in Tris-buffered saline containing 0.1% Tween-20 (TBST) for 1 h at room temperature. The membranes were then incubated overnight at 4 °C with the following primary antibodies: GPX4 (1:2000; Cat# A11243; Abclonal), FTH1 (1:5000; Cat# A19544; Abclonal), ACSL4 (1:1000; Cat#38493T; Cell Signaling Technology), xCT/SLC7A11 (1:1000; Cat# 28001T; Cell Signaling Technology), and β-actin (1:50000; Cat# 66009-1-Ig; Proteintech). After washing three times with TBST for 10 min each, the membranes were incubated with HRP-conjugated secondary antibodies, including goat anti-rabbit IgG (1:40000; Cat# EAB-1003; Elabscience) and goat anti-mouse IgG (1:40000; Cat# E-AB-1001; Elabscience), for 1 h at room temperature. Protein bands were visualized using an enhanced chemiluminescence reagent and imaged with a chemiluminescence imaging system. Band intensities were quantified using Image Lab and normalized to β-actin.

### Fe^2+^, glutathione (GSH), malondialdehyde (MDA) and superoxide dismutase (SOD) assay

Biochemical indices related to ferroptosis and oxidative stress were measured in the mPFC, hippocampus, and striatum using commercial assay kits. Briefly, tissues were homogenized in ice-cold PBS and centrifuged, and the resulting supernatants were collected for subsequent assays. Fe²⁺ levels were measured using a Tissue Iron Assay Kit (Cat#E-BC-K773-M; Elabscience, China) according to the manufacturer’s instructions. Total GSH and oxidized glutathione (GSSG) levels were assessed using a Total GSH/GSSG Detection Kit (Cat#E-BC-K097-M; Elabscience, China). MDA levels and SOD activity were measured using a Lipid Peroxidation MDA Assay Kit (Cat#E-BC-K025-M; Elabscience, China) and a Total SOD Assay Kit (Cat#E-BC-K020-M; Elabscience, China), respectively. Absorbance was measured using a microplate reader at the wavelengths specified in the manufacturer’s protocols. Concentrations or enzyme activities were calculated according to the standard curves or formulas provided by the manufacturers.

### HE and nissl staining

Mice were deeply anesthetized with pentobarbital sodium (70 mg/kg, intraperitoneally) and transcardially perfused with ice-cold phosphate-buffered saline (PBS) on PND24 to remove blood before tissue fixation. Brains were rapidly removed and post-fixed in 4% paraformaldehyde (PFA) at 4 °C for 24 h, followed by dehydration through a graded ethanol series (70%, 80%, 95%, and 100%), clearing in xylene, and embedding in paraffin. Coronal brain sections containing the mPFC, striatum, and hippocampus were cut at 5 μm thickness using a rotary microtome and mounted onto glass slides. For HE staining, sections were deparaffinized in xylene, rehydrated through descending ethanol gradients to distilled water, and stained using a commercial HE staining kit (C0105S; Beyotime Biotechnology, China) according to the manufacturer’s instructions. Hematoxylin staining was performed for 5 min, followed by rinsing and eosin counterstaining for 3 min. Slides were then dehydrated, cleared, and sealed with neutral resin. For Nissl staining, sections were deparaffinized, rehydrated, and incubated with Nissl staining solution (C0117; Beyotime Biotechnology, China) for 10 min at 37 °C. After thorough rinsing, sections were dehydrated, cleared, and coverslipped. The number of Nissl-positive neurons was quantified in the mPFC, striatum, and hippocampal CA1, CA3, and dentate gyrus (DG) regions using the Cell Counter plugin of ImageJ software (NIH, Bethesda, MD, USA). For each animal, three non-overlapping high-power fields were selected from each region of interest. Nissl-positive neurons with clear cell bodies and visible morphology were manually counted by an investigator blinded to group assignment. The mean value from all selected fields for each animal was used for subsequent statistical analysis.

### Transmission electron microscopy (TEM)

Mitochondrial ultrastructure was examined using TEM. Small tissue blocks approximately 1 mm³ were rapidly dissected from the mPFC, hippocampus, and striatum to minimize ultrastructural artifacts and preserve organelle integrity. The samples were immediately fixed in 2.5% glutaraldehyde electron microscopy fixative at 4 °C, followed by post-fixation with 1% osmium tetroxide. After fixation, samples were dehydrated through a graded ethanol series, embedded in epoxy resin, and polymerized at 60 °C for 48 h. Semi-thin Section  (1 μm) were stained with toluidine blue for preliminary region selection. Ultrathin sections of 60–90 nm were then cut, mounted on copper grids, stained with 2% uranyl acetate and lead citrate, and examined using a transmission electron microscope (Hitachi, HT7700). Mitochondrial abnormalities were evaluated based on morphological features, including mitochondrial shrinkage or swelling, reduced or disrupted cristae, increased membrane density, vacuolization, and outer membrane rupture.

### Reactive oxygen species (ROS) assay

ROS-related fluorescence in brain sections was detected using dihydroethidium staining (DHE; Cat# S0063; Beyotime Biotechnology, China). Frozen coronal brain sections of 8 μm thickness mounted on glass slides were incubated with DHE working solution diluted 1:200 in PBS at 37 °C for 30 min in the dark. The sections were then washed three times with PBS for 5 min each in the dark. After residual PBS was removed, the sections were mounted with antifade mounting medium containing DAPI (Cat# G1407-25ML; Servicebio, China) for nuclear counterstaining and kept at room temperature for 10 min in the dark. DHE fluorescence images of the mPFC, hippocampus, and striatum were captured using a fluorescence microscope (Zeiss, Germany) under identical exposure and acquisition settings for all groups. Mean fluorescence intensity within regions of interest in the mPFC, hippocampus, and striatum was quantified using ImageJ software after background subtraction by an investigator blinded to group assignment.

### Immunofluorescence (IF) staining

Mice were deeply anesthetized with pentobarbital sodium (70 mg/kg, intraperitoneally) and then transcardially perfused with ice-cold PBS on PND24. Brain hemispheres were harvested and post-fixed in 4% PFA at 4 °C for 24 h. The tissues were then cryoprotected in sucrose solution until they sank, embedded in optimal cutting temperature compound, and frozen for cryosectioning. Coronal brain sections were cut at a thickness of 10 μm using a cryostat and mounted onto glass slides for subsequent immunofluorescence staining. The fluorescence intensity of xCT/SLC7A11 and ACSL4 in the mPFC, hippocampus, and striatum was assessed in each group. Frozen sections were brought to room temperature, washed three times with PBS, and permeabilized and blocked with 0.2% Triton X-100 and 5% BSA for 30 min at room temperature. The sections were then incubated overnight at 4 °C with rabbit anti-SLC7A11 antibody (Cat# 26864-1-AP; Proteintech) and rabbit anti-ACSL4 antibody (Cat# R24265; Zenbio), respectively. After primary antibody incubation, the sections were equilibrated at room temperature for 30 min and washed three times with PBS. The sections were then incubated with the corresponding goat anti-rabbit IgG H&L Alexa Fluor 488 secondary antibody (Cat# ab150077; Abcam) at 37 °C for 1 h in the dark. After washing with PBS, the sections were mounted with antifade mounting medium containing DAPI. Fluorescence images of the prelimbic cortex (PL) and infralimbic area (ILA) of the mPFC, hippocampus, and striatum were captured using a fluorescence microscope (Leica Microsystems, Germany). All images were acquired and processed using identical parameters to ensure consistency and minimize bias. Fluorescence intensity was quantified using ImageJ software. The mean fluorescence intensity was calculated as integrated density (IntDen) divided by the selected area and expressed in arbitrary units.

### Statistical analysis

In this study, all statistical analyses were performed using SPSS statistical software (version 27.0) and GraphPad Prism 9.5. Behavioral assessments and image-based quantification were performed by investigators blinded to group assignment. Data normality was assessed using the Shapiro–Wilk test, and homogeneity of variance was evaluated using Levene’s test. For the three-chamber social interaction test, paired t-tests were used to compare within-subject differences in interaction time between S1 and E during the sociability phase, and between S2 and S1 during the social novelty phase. For comparisons among four groups, one-way analysis of variance (ANOVA) was performed, and Tukey’s multiple comparisons test was applied. All results are presented as mean ± SEM, with n representing the number of independent biological replicates. Statistical significant was defined as *P* < 0.05. The following significance levels were used: *p < 0.05, **p < 0.01, ***p < 0.001, and “ns” indicates no statistical difference.

## Result

### Prenatal VPA exposure induces sex differences of social deficits and anxiety-like behaviors

**Fig. 1:**
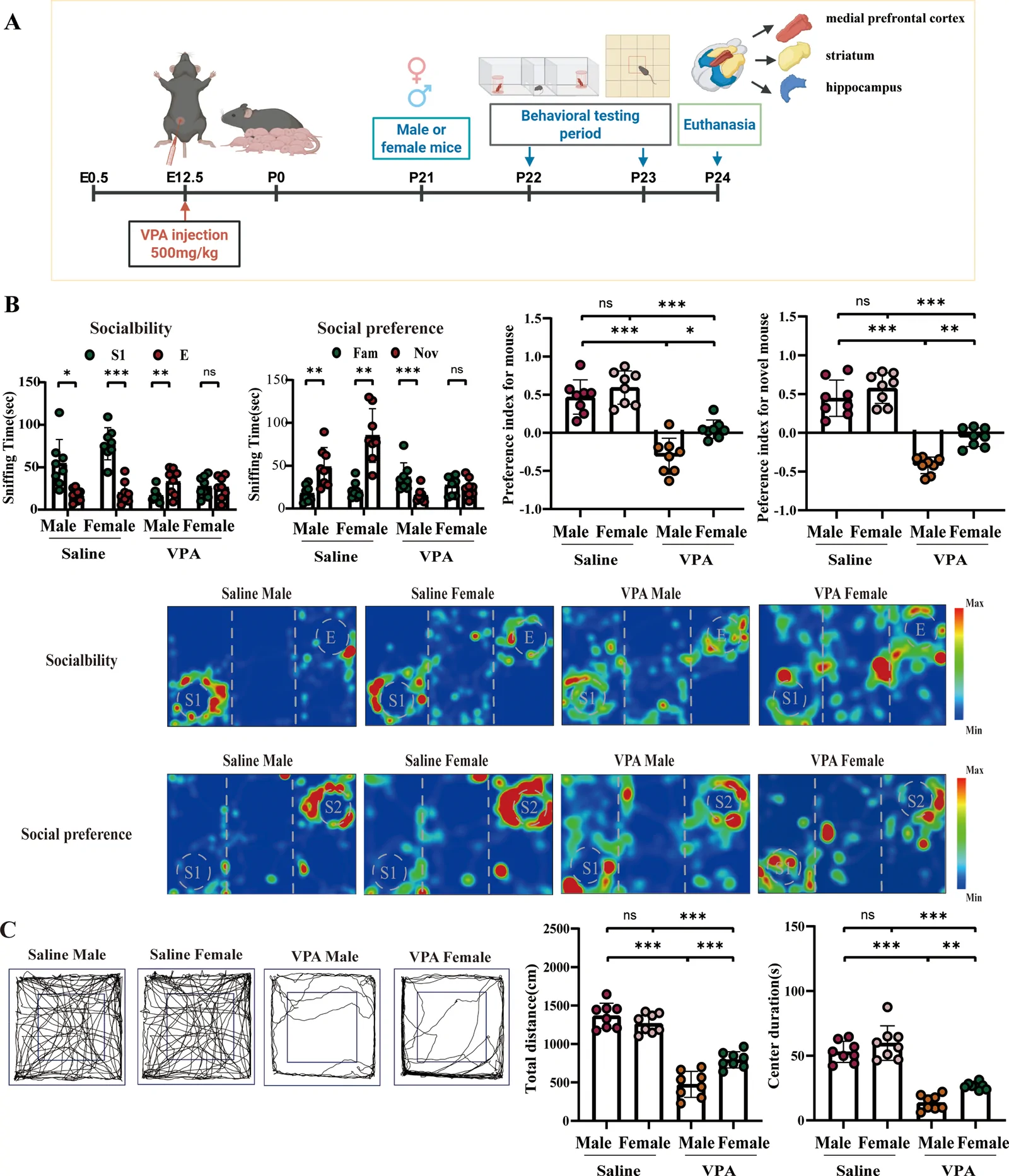
Prenatal VPA exposure induces sex differences impairments in social behavior and anxiety-like behavior in mice. **A** An outline of the experimental process for performing behavior measures. **B** Representative traces of 3-CT (E: Empty, S1: stranger 1, S2: stranger 2). Statistical graph of sociability and social novelty test. *n* = 8 per group. **C** Representative traces of OFT. Statistical graph of the percent of time spent in the center and total distance. *n* = 8 per group. Values was presented as mean ± SEM and analyzed by one-way ANOVA and two-tailed paired t-tests. *ns* > 0.05, *p < 0.05, **p < 0.01, ***p < 0.001

As described previously, the ASD-like mouse model was established by intraperitoneal injection of VPA into pregnant dams on E12.5, a procedure known to induce social behavioral abnormalities, molecular alterations, neuroanatomical changes, and immune dysregulation [40, 41]. Following completion of the behavioral tests, general developmental and gross neuroanatomical parameters, including body weight, body length, tail length, and brain wet weight, were evaluated at PND24. Compared with their respective saline-exposed male and female mice, both VPA-exposed male and female mice showed significantly lower body weight. Tail length was significantly reduced in VPA-exposed female mice but did not differ significantly between VPA-exposed and saline-exposed male mice. No significant differences in body length or brain wet weight were observed between VPA-exposed and saline-exposed mice of either sex (Supplementary Fig. S1A–D). These findings suggest that prenatal VPA exposure may influence somatic growth in offspring. Behavioral tests were performed from PND22 to PND23 to determine whether prenatal VPA exposure induced sex differences in social behavior, locomotor activity, and anxiety-like behavior (Fig. 1). In the 3-CT, during the sociability phase, saline-exposed male and female mice spent more time sniffing the S1 than the E, indicating normal sociability. In contrast, VPA-exposed male mice failed to show a clear preference for S1 over E and showed relatively increased sniffing of the empty cage, suggesting impaired sociability. VPA-exposed female mice spent comparable amounts of time sniffing S1 and E, indicating attenuated sociability, although this impairment appeared less pronounced than that observed in VPA-exposed males. Consistently, VPA exposure significantly reduced the sociability index, with VPA-exposed males showing a lower sociability index than VPA-exposed females. During the social novelty phase, saline-exposed mice showed a preference for the S2 over the S1, suggesting intact social novelty preference. In contrast, VPA-exposed male mice showed increased sniffing time toward S1 and failed to preferentially investigate S2, indicating impaired social novelty preference. VPA-exposed female mice spent nearly comparable amounts of time sniffing S1 and S2, suggesting a milder disruption of social novelty preference. Consequently, the social novelty index was significantly lower in VPA-exposed mice than in saline-exposed mice. Collectively, E12.5 VPA exposure reduced both sociability and social novelty preference indices, with males showing greater impairments than females (Fig. 1B). To evaluate locomotor activity and anxiety-like behavior, mice were subjected to the OFT. Representative movement trajectories showed broad exploration in saline-exposed male and female mice. VPA-exposed mice displayed reduced exploratory activity, with trajectories mainly restricted to the peripheral zone. Quantitative analysis showed that VPA exposure reduced total distance traveled, indicating decreased locomotor activity. VPA-exposed mice also spent less time in the center zone, consistent with increased anxiety-like behavior. Notably, this center-zone avoidance was more pronounced in VPA-exposed males than in VPA-exposed females, suggesting stronger anxiety-like alterations in males (Fig. 1C). Taken together, prenatal VPA exposure induces social deficits, reduced locomotor activity, and anxiety-like behavioral alterations in adolescent offspring, with more pronounced changes observed in VPA-exposed male offspring than in VPA-exposed female offspring, indicating sex differences in behavioral responses to prenatal VPA exposure.

### Prenatal VPA exposure is associated with prominent neuronal alterations in the mPFC

**Fig. 2:**
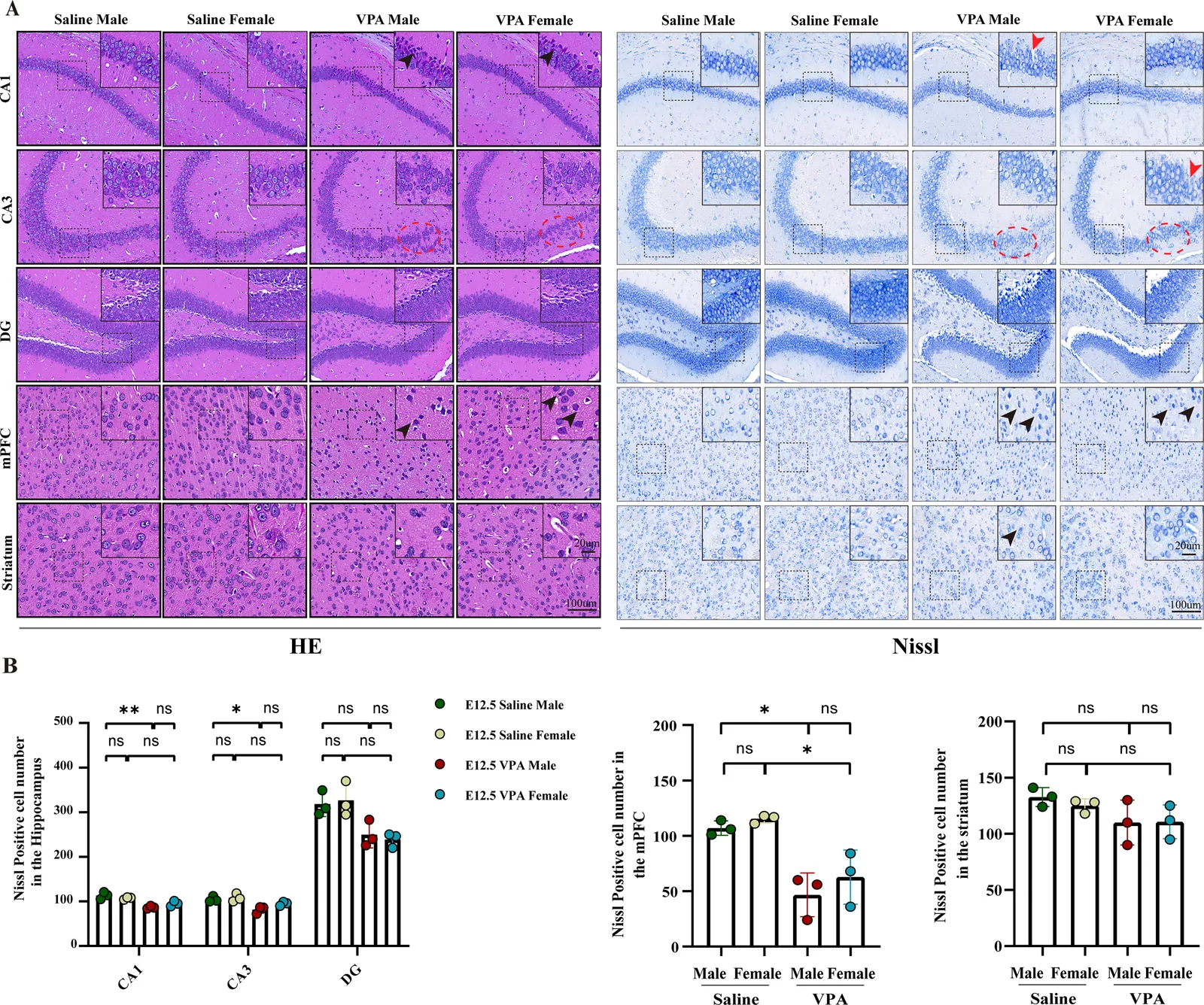
Prenatal VPA Exposure is Associated with Region-Specific Neuronal Alterations in the mPFC, Hippocampus, and Striatum. **A** Observation of the morphology of brain nerve cells in the mPFC, hippocampus and striatum by HE staining. Scale bar = 100 (raw)/20 (zoom) µm. Observation of the morphology of neurons in the mPFC, hipppcampus and striatum by Nissl staining. Scale bar = 100 (raw)/20 (zoom) µm. *n* = 3 per group. The red arrow represents rarefaction-like changes, the black arrow represents cytoplasmic vacuolization, nuclear pyknosis and poorly defined nucleoli. **B** Statistical graph of nissl body in the mPFC, hippocampus and striatum. *n* = 3 per group. Values was presented as mean ± SEM and analyzed by one-way ANOVA. *ns* > 0.05, *P < 0.05, **P < 0.01, ***P < 0.001. HE, hematoxylin-eosin; SEM, standard error of mean

To evaluate brain morphological alterations associated with ASD-like behaviors following prenatal VPA exposure at E12.5, we performed HE and Nissl staining in the mPFC, hippocampus, and striatum of male and female mice. The mPFC is related to social and executive functions. The hippocampus is important for learning, memory, and social memory. The striatum is involved in reward processing, motor regulation, and repetitive behaviors. These regions may therefore provide useful information about brain developmental disorders associated with VPA-induced ASD-like abnormalities. HE staining showed clear histopathological changes in the mPFC of VPA-exposed male mice. Compared with saline-exposed male mice, VPA-exposed male mice showed focal rarefaction-like changes, cytoplasmic vacuolization, nuclear pyknosis, and poorly defined nucleoli. Similar structural abnormalities were also observed in the mPFC of VPA-exposed female mice. Histological alterations were also observed in the hippocampus of both male and female mice following VPA exposure, although these changes appeared less prominent than those observed in the mPFC. In contrast, no obvious histological abnormalities were observed in the striatum (Fig. 2A). Nissl staining further supported these histological observations. In the mPFC, both male and female VPA-exposed mice exhibited significantly fewer Nissl-positive neurons than their respective saline-exposed controls. However, no significant difference in the number of Nissl-positive neurons was detected between VPA-exposed male and female mice. In the hippocampus, VPA-exposed male mice showed a significant reduction in Nissl-positive neuronal number compared with saline-exposed male mice, whereas no significant difference was observed between VPA-exposed and saline-exposed female mice. In the striatum, no significant differences in Nissl-positive neuronal number were detected among the groups (Fig. 2B). Collectively, these findings indicate that prenatal VPA exposure was associated with region-specific neurohistological alterations, with the most prominent changes observed in the mPFC. Notably, mPFC alterations were detected in both male and female offspring, without a significant difference in Nissl-positive neuronal number between the VPA-exposed sexes.

### Prenatal VPA exposure induces region-specific ferroptosis-related alterations with sex differences in the mPFC

Recent studies have suggested that ferroptosis may be involved in neuropathological alterations observed in VPA-induced ASD-like models [19, 29]. To further investigate the mechanisms underlying neuronal injury and sex differences behavioral vulnerability following prenatal VPA exposure, we assessed key ferroptosis-related features, including iron accumulation, lipid peroxidation, oxidative stress, and dysregulated glutathione metabolism, in the mPFC, hippocampus, and striatum.

#### E12.5 VPA exposure induces more pronounced ferroptosis-related alterations in the mPFC of male offspring

**Fig. 3:**
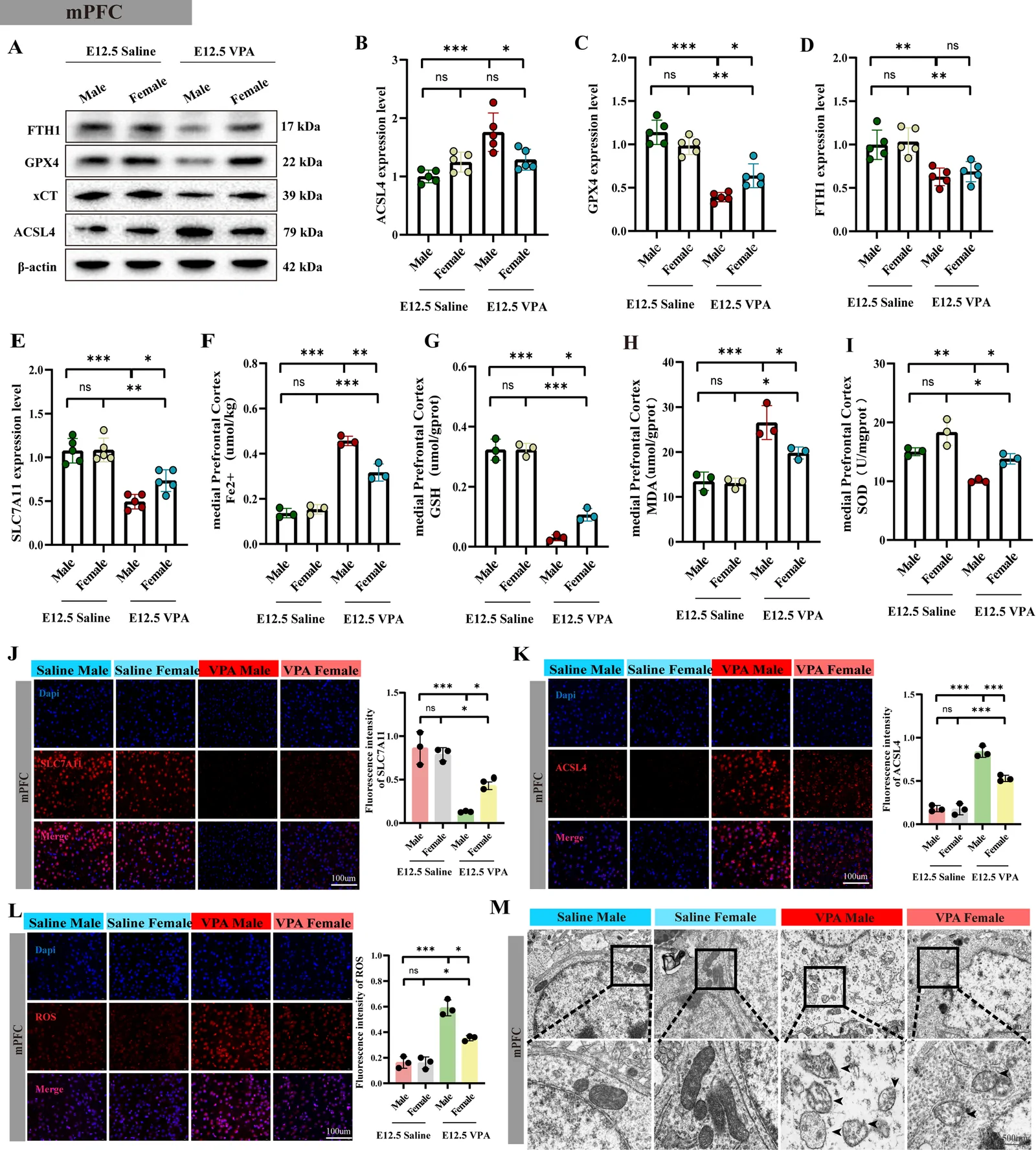
E12.5 VPA exposed induces male-biased ferroptosis-associated alterations in the mPFC. **A** Representative protein bands of GPX4, SLC7A11/xCT, ACSL4 and FTH1 in mPFC. **B-E** Statistical graph of protein levels of GPX4, SLC7A11/xCT, ACSL4 and FTH1. *n* = 5 per group. **F** Content of ferrous iron in the P24 mPFC. *n* = 3 per group. **G** GSH concentration and the ratio of GSH and GSSH in the P24 mPFC measured. *n* = 3 per group. **H** The MDA level in the P24 mPFC. *n* = 3 per group. **I** The SOD concentration in the P24 mPFC. *n* = 3 per group. **J** Representative immunofluorescence images of SLC7A11 (red) and DAPI (blue) in mPFC. Scale bar = 100 μm. Mean fluorescence intensity of SLC7A11/xCT, *n* = 3 per group. **K** Representative immunofluorescence images of ACSL4 (red) and DAPI (blue) in mPFC. Scale bar = 100 μm. Mean fluorescence intensity of ACSL4, *n* = 3 per group. **L** Representative immunofluorescence images of ROS (red) and DAPI (blue) in mPFC. Scale bar = 100 μm. Mean fluorescence intensity of ACSL4, *n* = 3 per group. **M** Electron microscope of mitochondrial morphology in the P24 mPFC. The second row represents a partial enlargement of the first row, respectively. Scale bar = 2 μm (raw) / 500 nm (zoom). Values was presented as mean ± SEM and analyzed by one-way ANOVA. *ns* > 0.05, *p < 0.05, **p < 0.01, ***p < 0.001

In the mPFC, VPA-exposed male mice showed significantly elevated ferrous iron (Fe²⁺) levels compared with saline-exposed males, accompanied by decreased ferritin heavy chain 1 (FTH1) expression (Fig. 3A, D, F). This pattern suggests that VPA exposure disrupts iron homeostasis in the mPFC. Notably, VPA-exposed male mice exhibited more pronounced disturbances in iron-related indices than VPA-exposed female mice, indicating a sex differences in iron homeostatic disruption. We next examined oxidative stress and lipid peroxidation, two key processes associated with ferroptosis. Compared with saline-exposed male mice, VPA-exposed male mice showed significantly increased ROS levels (Fig. 3L), suggesting enhanced oxidative stress. Consistently, lipid peroxidation-related indicators were also elevated, as shown by increased MDA content and upregulated acyl-CoA synthetase long-chain family member 4 (ACSL4), a key enzyme involved in lipid remodeling and ferroptosis-related lipid peroxidation (Fig. 3A, B, H, K). In parallel, antioxidant defenses were impaired, as indicated by reduced SOD activity and decreased GSH levels (Fig. 3I, J). These changes were accompanied by downregulation of SLC7A11/xCT and glutathione peroxidase 4 (GPX4), two important components of the antioxidant system involved in GSH-dependent detoxification of lipid peroxides (Fig. 3A, C, E, G). Collectively, these molecular and biochemical changes indicate that prenatal VPA exposure induces ferroptosis-related oxidative and lipid peroxidation alterations in the mPFC. Importantly, these alterations were more pronounced in males than in females, suggesting sex differences in ferroptosis-related alterations in the mPFC. TEM further revealed mitochondrial ultrastructural abnormalities in the mPFC of VPA-exposed male mice, including disrupted cristae, vacuolization, and shrunken, rounded mitochondrial morphology (Fig. 3M). Together with the observed increase in Fe²⁺, enhanced lipid peroxidation, impaired antioxidant defense, and reduced GPX4 and SLC7A11/xCT expression, these mitochondrial changes are consistent with a ferroptosis-like phenotype. Overall, these findings suggest that prenatal VPA exposure induces ferroptosis-related alterations in the mPFC, with more pronounced changes observed in male offspring than in female offspring.

#### E12.5 VPA exposure induces ferroptosis-related alterations in the hippocampus without obvious sex differences

**Fig. 4:**
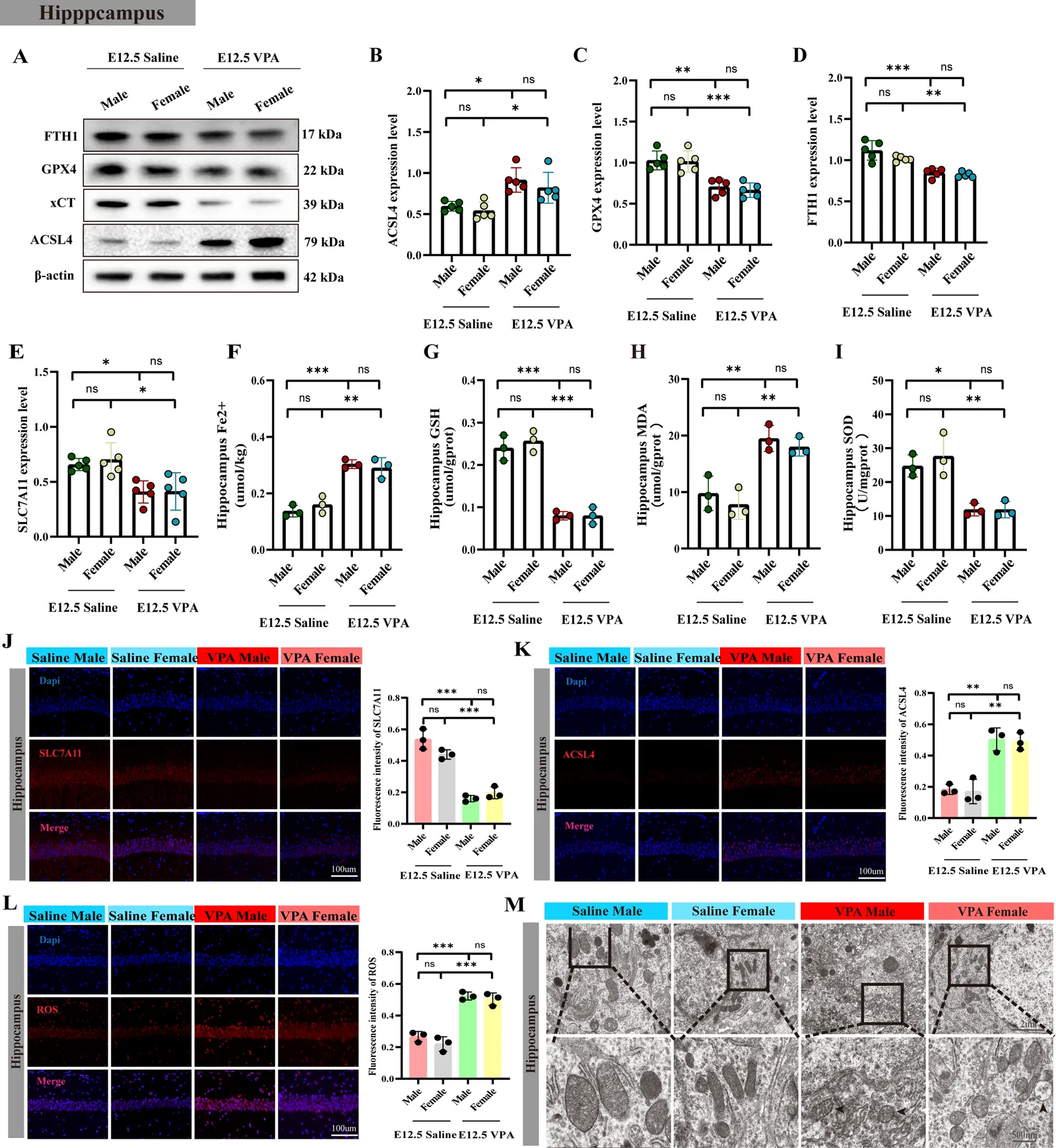
E12.5 VPA induces ferroptosis-related signatures in the hippocampus without sex differences. **A** Representative protein bands of GPX4, SLC7A11/xCT, ACSL4 and FTH1 in hippocampus. **B-E** Statistical graph of protein levels of GPX4, SLC7A11/ xCT, ACSL4 and FTH1. *n* = 5 per group. **F** Content of ferrous iron in the P24 hippocampus. *n* = 3 per group. **G** GSH concentration and the ratio of GSH and GSSH in the P24 hippocampus measured. *n* = 3 per group. **H** The MDA level in the P24 hippocampus. **I** The SOD concentration in the P24 hippocampus. **J** Representative immunofluorescence images of SLC7A11 (red) and DAPI (blue) in hippocampus. Scale bar = 100 μm. Mean fluorescence intensity of SLC7A11, *n* = 3 per group. **K** Representative immunofluorescence images of ACSL4 (red) and DAPI (blue) in hippocampus. Scale bar = 100 μm. Mean fluorescence intensity of ACSL4, *n* = 3 per group. **L** Representative immunofluorescence images of ROS (red) and DAPI (blue) in hippocampus. Scale bar = 100 μm. Mean fluorescence intensity of ACSL4, *n* = 3 per group. **M** Electron microscope of mitochondrial morphology in the P24 hippocampus. The second row represents a partial enlargement of the first row, respectively. Scale bar = 2 μm (raw) / 500 nm (zoom). Values was presented as mean ± SEM and analyzed by one-way ANOVA. *ns* > 0.05, *p < 0.05, **p < 0.01, ***p < 0.001

In the hippocampus, VPA-exposed male mice showed significantly decreased protein levels of FTH1, GPX4, and SLC7A11/xCT compared with saline-exposed male mice, whereas ACSL4 expression was significantly increased (Fig. 4A–E, J, K). In parallel, Fe²⁺, ROS, and MDA levels were significantly elevated (Fig. 4F, H, L), whereas GSH levels and SOD activity were significantly reduced (Fig. 4G, I). These findings suggest that prenatal VPA exposure induces iron dyshomeostasis, enhanced lipid peroxidation, and impaired antioxidant defense in the male hippocampus, collectively consistent with ferroptosis-related molecular and biochemical alterations. TEM analysis further revealed mitochondrial ultrastructural abnormalities in the hippocampus of VPA-exposed male mice, characterized by shrunken and rounded mitochondria, as well as fragmented or disrupted cristae (Fig. 4M). In female mice, VPA exposure produced changes in a generally similar direction across ferroptosis-related indices. However, within the VPA-exposed group, no significant differences were detected between males and females for these measures. These results indicate that prenatal VPA exposure is associated with ferroptosis-related alterations in the hippocampus, with no significant sex differences detected across the ferroptosis-related measures examined.

#### E12.5 VPA exposure induces ferroptosis-related alterations in the striatum without obvious sex differences

**Fig. 5:**
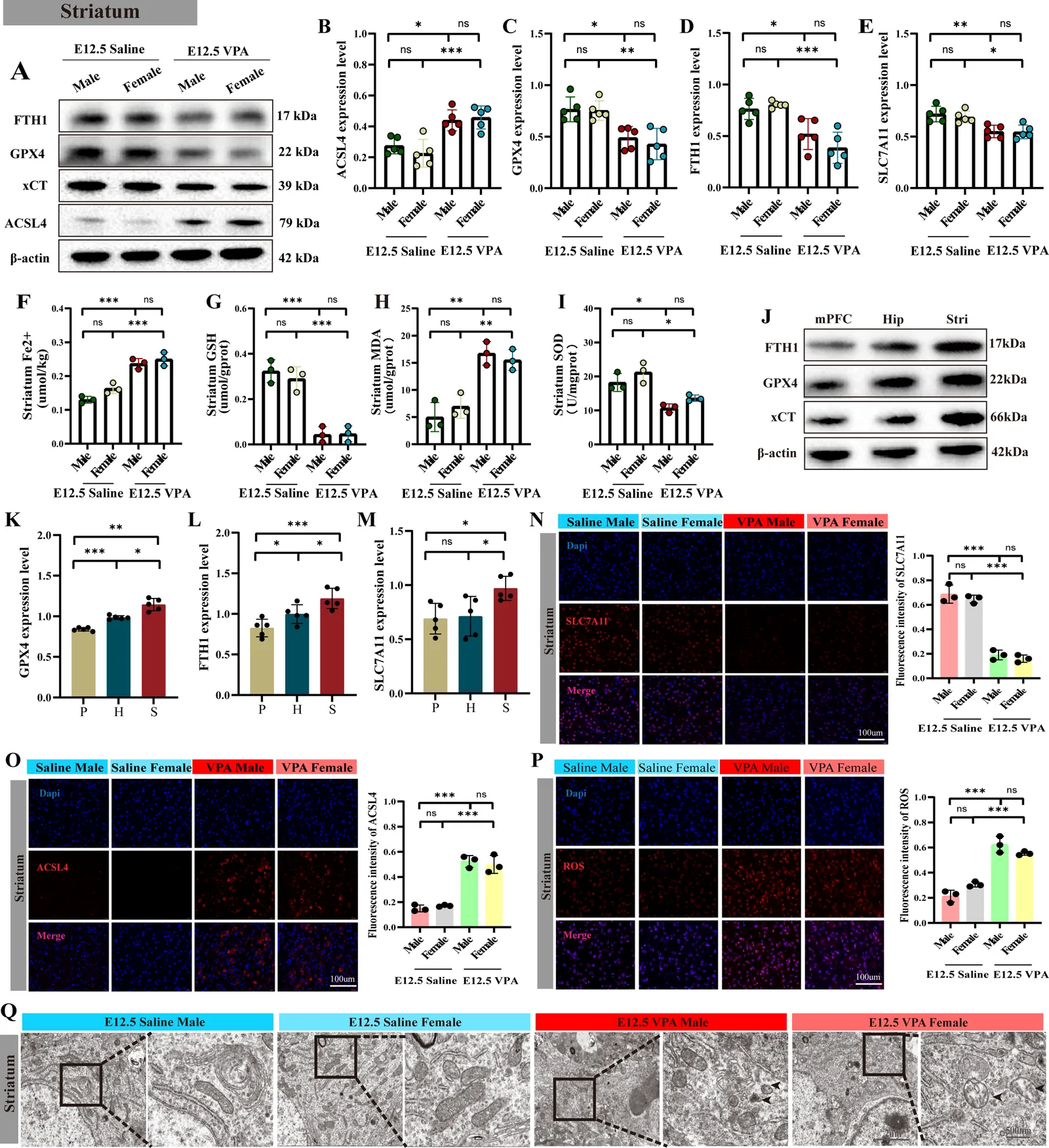
E12.5 VPA induces ferroptosis-related alterations in the striatum without sex differences. **A** Representative protein bands of GPX4, SLC7A11/xCT, ACSL4 and FTH1 in striatum. **B-E** Statistical graph of protein levels of GPX4, SLC7A11/xCT, ACSL4 and FTH1. *n* = 5 per group. **F** Content of ferrous iron in the P24 striatum. *n* = 3 per group. **G** GSH concentration and the ratio of GSH and GSSH in the P24 striatum measured. *n* = 3 per group. **H** The MDA level in the P24 striatum. **I** The SOD concentration in the P24 striatum. **J** Representative protein bands of GPX4, SLC7A11/xCT and FTH1 in mPFC, hippocampus and striatum. **K-M** Statistical graph of protein levels of GPX4, SLC7A11/xCT and FTH1. *n* = 5 per group. **N** Representative immunofluorescence images of SLC7A11/xCT (red) and DAPI (blue) in striatum. Scale bar = 100 μm. Mean fluorescence intensity of SLC7A11/xCT, *n* = 3 per group. **O** Representative immunofluorescence images of ACSL4 (red) and DAPI (blue) in striatum. Scale bar = 100 μm. Mean fluorescence intensity of ACSL4, *n* = 3 per group. **P** Representative immunofluorescence images of ROS (red) and DAPI (blue) in striatum. Scale bar = 100 μm. Mean fluorescence intensity of ROS, *n* = 3 per group. **Q** Electron microscope of mitochondrial morphology in the P24 striatum. Scale bar = 2 μm (raw) / 500 nm (zoom). Values was presented as mean ± SEM and analyzed by one-way ANOVA. *ns* > 0.05, *p < 0.05, **p < 0.01, ***p < 0.001

In the striatum, VPA-exposed male mice exhibited a ferroptosis-related molecular and biochemical profile similar to that observed in the hippocampus. Compared with saline-exposed male mice, VPA-exposed male mice showed significantly decreased protein levels of FTH1, GPX4, and SLC7A11/xCT, whereas ACSL4 expression was significantly increased (Fig. 5A-E, N, O). In parallel, Fe²⁺, ROS, and MDA levels were significantly elevated (Fig. 5F, H, P), whereas GSH levels and SOD activity were significantly reduced (Fig. 5G, I). These results indicate that prenatal VPA exposure induces iron dyshomeostasis, lipid peroxidation, and antioxidant imbalance in the striatum. Consistently, TEM revealed mitochondrial ultrastructural damage in the striatum of VPA-exposed male mice, including shrunken and rounded morphology, fragmented or disrupted cristae, and prominent vacuolization (Fig. 5Q). Female mice showed changes in the same general direction. However, no significant sex differences were detected within the VPA-exposed group across these indices. These findings suggest that VPA-associated ferroptosis-related alterations in the striatum did not differ significantly between male and female mice.

Overall, prenatal VPA exposure induced ferroptosis-related alterations in the mPFC, hippocampus, and striatum, characterized by disrupted iron homeostasis, enhanced oxidative stress and lipid peroxidation, and impaired antioxidant balance. Sex differences were most evident in the mPFC, where these changes were more pronounced in males than in females. In contrast, no significant sex differences were detected in the hippocampus or striatum under VPA exposure. Cross-region comparisons in VPA-exposed male mice further revealed region-specific alterations in ferroptosis-related protein expression across mPFC, hippocampus, and striatum. Among these regions, the mPFC showed the most pronounced reduction in FTH1, GPX4, and SLC7A11/xCT levels (Fig. 5J–M). Together, these findings suggest that prenatal VPA exposure induces region-specific ferroptosis-related alterations, with sex-related differences particularly evident in the mPFC.

### Prenatal and postnatal VPA exposure produce comparable autism-like behavioral deficits in male mice

**Fig. 6:**
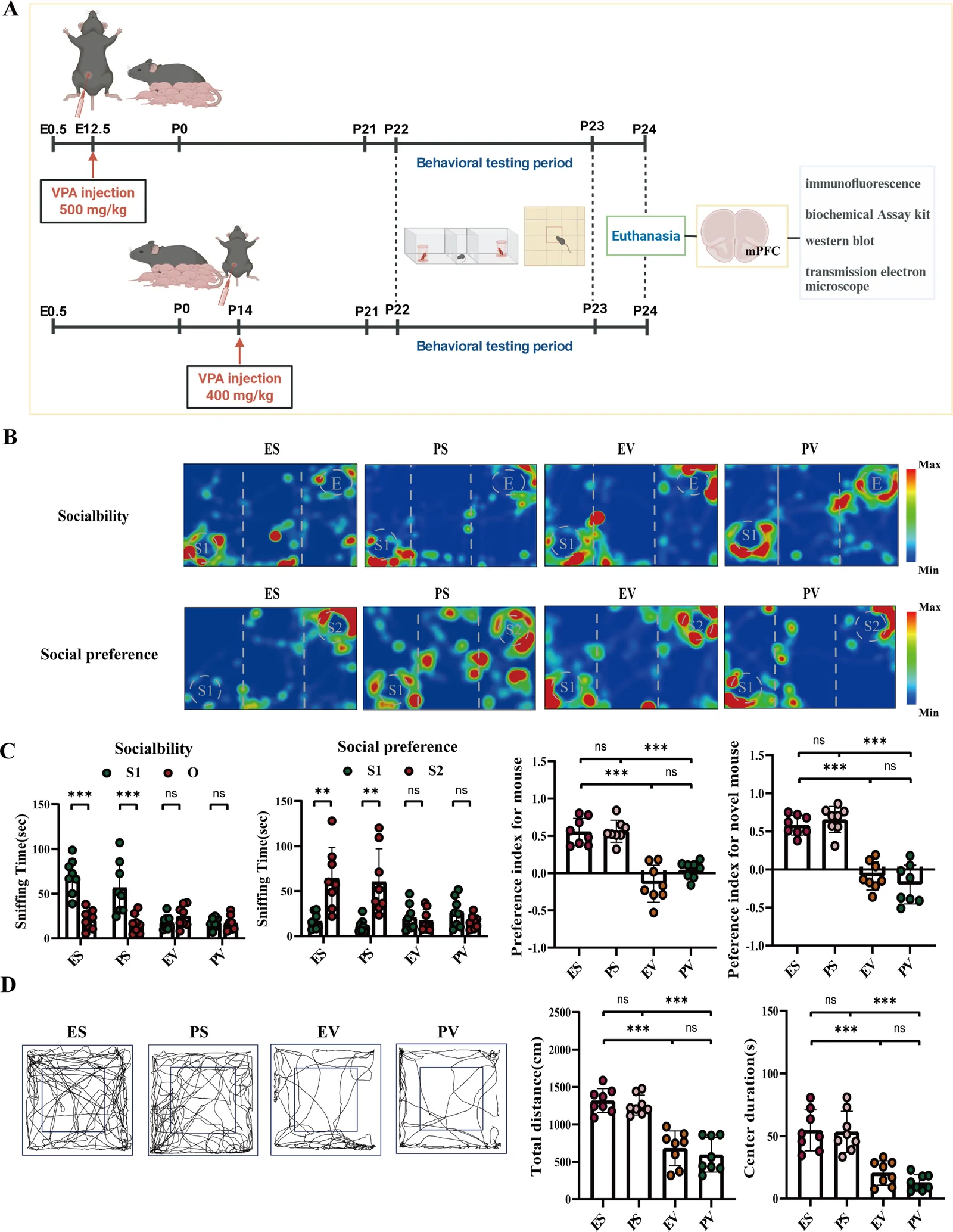
Prenatal and postnatal VPA exposure induces comparable social deficits and anxiety-like behavior in mice. **A** An outline of the experimental process for performing behavior measures. **B** Representative traces of three-chamber social interaction test (E: Empty, S1: stranger 1, S2: stranger 2). Statistical graph of sociability and social novelty test. *n* = 8 per group. **C** Representative traces of open-field test. Statistical graph of the percent of time spent in the center and total distance. *n* = 8 per group. Values was presented as mean ± SEM and analyzed by one-way ANOVA. *ns* > 0.05, *p < 0.05, **p < 0.01, ***p < 0.001. ES: E12.5 Saline-exposed. PS: PND14 Saline-exposed. EV: E12.5 VPA-exposed. PV: PND14 VPA-exposed

Together, these findings suggest that male mice could be more susceptible to VPA-induced ferroptosis-related alterations, particularly in the mPFC. To compare the effects of VPA exposure across distinct developmental windows, male mice were exposed to VPA either prenatally at E12.5 or postnatally at PND14, followed by systematic assessment of developmental parameters and ASD-like behavioral changes. This only male design was adopted to focus on the more vulnerable sex identified in the preceding experiments while minimizing potential confounding effects related to sex-by-window interactions. Following completion of the behavioral tests, body weight, body length, tail length, and brain wet weight were evaluated at PND24. Compared with their respective saline-exposed controls, both E12.5 and PND14 VPA-exposed mice showed significantly reduced body weight and tail length. Body length was significantly reduced in E12.5 VPA-exposed mice but did not differ significantly between PND14 VPA-exposed and saline-exposed mice. In contrast, brain wet weight was significantly reduced in PND14 VPA-exposed mice but did not differ significantly between E12.5 VPA-exposed and saline-exposed mice (Supplementary Fig. S1E-H). These findings suggest that VPA exposure during both prenatal and early postnatal developmental periods is associated with impaired somatic growth. In the 3-CT, during the sociability phase, both E12.5 and PND14 VPA-exposed mice spent significantly more time investigating the S1 than the E (Fig. 6B, C). However, compared with their respective saline-exposed controls, the sociability index was significantly reduced in both prenatal and postnatal VPA-exposed mice, indicating attenuated sociability following VPA exposure. Notably, no significant difference in the sociability index was detected between E12.5 and PND14 VPA-exposed groups. During the social novelty phase, both prenatal and postnatal VPA-exposed mice spent comparable amounts of time investigating S1 and the S2, indicating impaired social novelty preference. Consistently, the social novelty index was significantly decreased in both E12.5 and PND14 VPA-exposed mice compared with their respective saline-exposed controls, with no significant difference between the two VPA exposure windows. These findings suggest that VPA exposure, whether administered prenatally at E12.5 or postnatally at PND14, induces broadly comparable social behavioral deficits in male mice. In the OFT, both E12.5 and PND14 VPA-exposed mice showed significant reductions in total distance traveled and time spent in the center zone compared with their respective saline-exposed controls (Fig. 6D). No significant differences were observed between the two VPA-exposed groups in either parameter. These results suggest that VPA exposure at either developmental window reduces spontaneous locomotor activity and is associated with anxiety-like behavioral alterations. However, reduced center-zone exploration should be interpreted in the context of decreased overall locomotor activity. Overall, prenatal and postnatal VPA exposure produced broadly comparable ASD-like behavioral abnormalities in male mice.

### Prenatal and postnatal VPA exposure induce comparable ferroptosis-related mPFC injury in male mice

The above findings suggest that VPA-induced ASD-like behaviors and sex differences in vulnerability could be associated with ferroptosis-related neuronal injury in the mPFC. Given that both prenatal and early postnatal VPA exposure have been reported to induce ASD-like behavioral abnormalities, and that VPA-induced neurodevelopmental alterations may vary depending on the developmental stage of exposure, we next compared ferroptosis-related alterations between prenatal and postnatal VPA exposure windows. Because male mice showed more pronounced behavioral deficits and ferroptosis-related alterations in the preceding experiments, only male mice were included in the exposure-window comparison to focus on the more vulnerable sex identified above and to avoid additional sex-by-window interactions. We therefore assessed ferroptosis-related features, including iron accumulation, lipid peroxidation, glutathione metabolic dysfunction, and mitochondrial ultrastructural abnormalities, in the mPFC of male mice exposed to VPA at E12.5 or PND14.

**Fig. 7:**
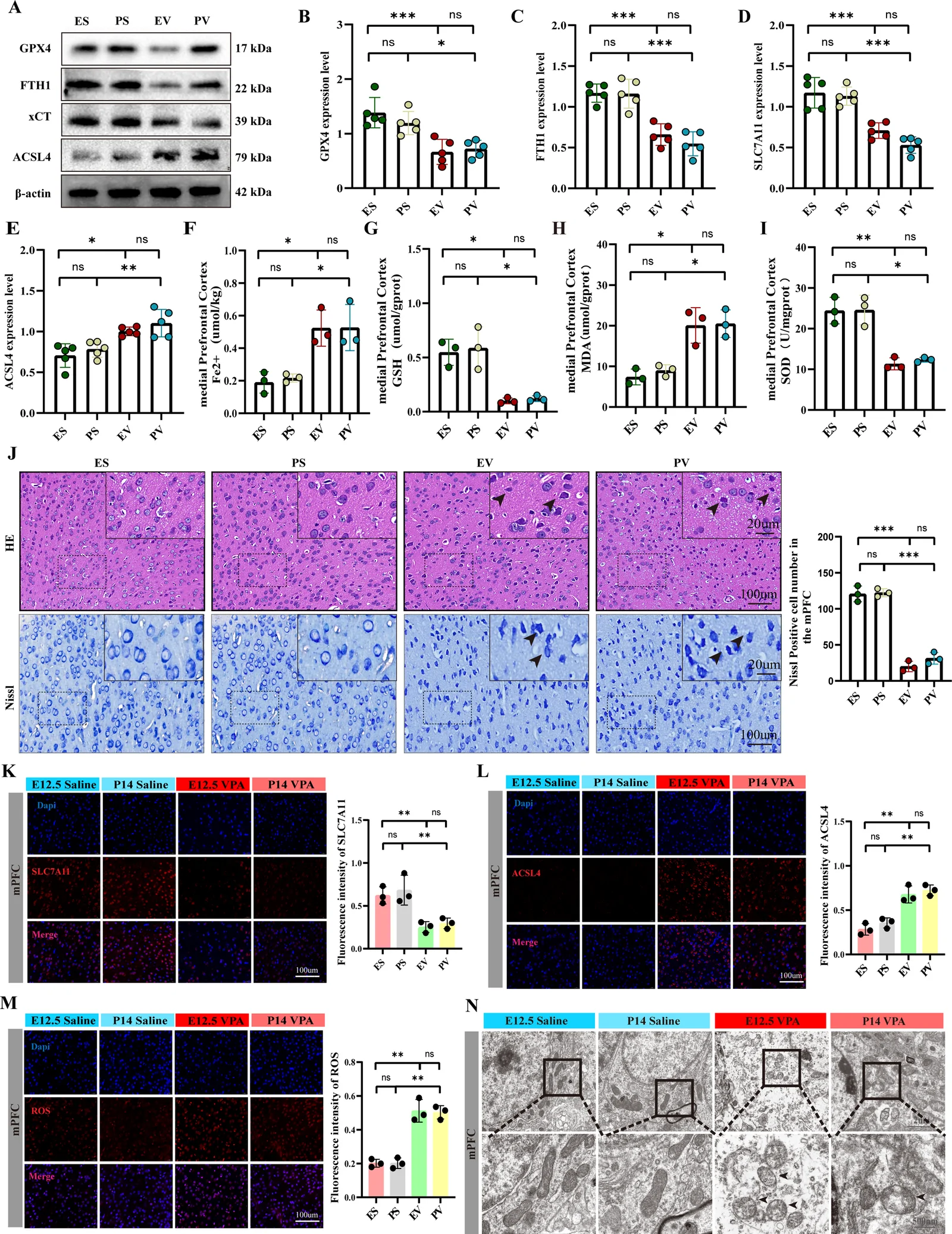
Comparable Ferroptosis-Associated alteration in the mPFC Following E12.5 and PND14 VPA Exposure. **A** Representative protein bands of GPX4, SLC7A11/xCT, ACSL4 and FTH1 in mPFC. **B-E** Statistical graph of protein levels of GPX4, SLC7A11/ xCT, ACSL4 and FTH1. *n* = 5 per group. **F** Content of ferrous iron in the P24 mPFC. *n* = 3 per group. **G** GSH concentration and the ratio of GSH and GSSH in the P24 mPFC measured. *n* = 3 per group. **H** The MDA level in the P24 mPFC. *n* = 3 per group. **I** The SOD concentration in the P24 mPFC. *n* = 3 per group. **J** Observation of the morphology of brain nerve cells in the mPFC by HE staining. Scale bar = 100 μm. The second row shows Nissl staining of neuronal morphology in the mPFC by. Scale bar = 100 (raw)/20 (zoom) µm. The black arrow represents cytoplasmic vacuolization, nuclear pyknosis and poorly defined nucleoli. Statistical graph of nissl body in the mPFC. *n* = 3 per group. **K** Representative immunofluorescence images of SLC7A11 (red) and DAPI (blue) in mPFC. Scale bar = 100 μm. Mean fluorescence intensity of SLC7A11, *n* = 3 per group. **L** Representative immunofluorescence images of ACSL4 (red) and DAPI (blue) in mPFC. Scale bar = 100 μm. Mean fluorescence intensity of ACSL4, *n* = 3 per group. **M** Representative immunofluorescence images of ROS (red) and DAPI (blue) in mPFC. Scale bar = 100 μm. Mean fluorescence intensity of ROS, *n* = 3 per group. **N** Electron microscope of mitochondrial morphology in the P24 mPFC. The second row represents a partial enlargement of the first row, respectively. Values was presented as mean ± SEM and analyzed by one-way ANOVA. ns > 0.05, *p < 0.05, **p < 0.01, ***p < 0.001

Compared with the prenatal saline group, prenatal VPA exposure induced marked neuropathological damage in the mPFC, as shown by HE and Nissl staining. These alterations included rarefaction-like changes, vacuolar degeneration, nuclear pyknosis, disorganized neuronal arrangement, and reduced Nissl staining, indicating neuronal structural damage (Fig. 7J). To further examine whether ferroptosis-related processes were associated with VPA-induced mPFC injury, we assessed molecular, biochemical, and ultrastructural features related to ferroptosis. Following prenatal VPA exposure, Fe²⁺ accumulation was significantly increased in the mPFC (Fig. 7F), accompanied by a significant reduction in FTH1 protein levels (Fig. 7A, C), suggesting disrupted iron homeostasis and increased labile iron burden. We next examined oxidative stress and lipid peroxidation, two key processes associated with ferroptosis. ROS-related fluorescence intensity was significantly increased after prenatal VPA exposure (Fig. 7M). Consistently, the level of MDA, a major end-product of lipid peroxidation, was significantly elevated (Fig. 7H). In parallel, ACSL4 was markedly upregulated (Fig. 7A, E, L). These findings indicate that prenatal VPA exposure enhances oxidative stress and lipid peroxidation in the mPFC. Furthermore, the antioxidant defense system was substantially impaired after prenatal VPA exposure. The protein levels of xCT/SLC7A11 and GPX4, as well as GSH levels and SOD activity, were significantly reduced (Fig. 7A, B, D, G, I, K). these results suggest that prenatal VPA exposure compromises anti-ferroptotic defense mechanisms in the mPFC. Consistent with these molecular and biochemical alterations, TEM revealed mitochondrial ultrastructural abnormalities after prenatal VPA exposure, including mitochondrial shrinkage, rounded morphology, and disorganized or absent cristae (Fig. 7N). Together, these findings support the presence of ferroptosis-related mPFC alterations after prenatal VPA exposure. Compared with the postnatal saline group, PND14 VPA exposure induced a similar pattern of ferroptosis-related alterations in the mPFC. Specifically, PND14 VPA exposure was associated with increased Fe²⁺ accumulation, reduced FTH1 expression, enhanced ROS fluorescence, elevated MDA levels, increased ACSL4 expression, and impaired antioxidant defense, as reflected by decreased xCT/SLC7A11, GPX4, GSH, and SOD levels. TEM analysis also revealed mitochondrial ultrastructural abnormalities following postnatal VPA exposure. These changes were generally consistent with those observed after prenatal VPA exposure. Direct comparison between the E12.5 and PND14 VPA-exposed groups revealed no statistically detectable differences in these ferroptosis-related indices. These finding indicate that both prenatal and postnatal VPA exposure can induce ferroptosis-related alterations in male mPFC. The comparable pattern of these alterations following E12.5 and PND14 VPA exposure suggests that ferroptosis-related changes in the mPFC may represent a shared molecular feature associated with VPA-induced ASD-like phenotypes across different developmental exposure windows.

## Discussion

The prenatal VPA-exposed animal model is extensively utilized in ASD research because of its clinical relevance and construct validity, and it reproduces a range of ASD-like behavioral and neurodevelopmental abnormalities [42–44]. However, most studies using this model have predominantly focused on males, and relatively few have systematically examined sex differences in behavioral phenotypes and brain molecular alterations. Clinically, ASD occurrence after in utero VPA exposure has been reported to be approximately two-fold higher in males than in females [43], although another study reported a nearly equal prevalence between the two sexes [44]. The latter study, however, analyzed exposure to antiepileptic drugs in general rather than VPA specifically. Therefore, investigating sex differences in VPA-exposed animal models may provide further insight into sex-dependent phenotypic characteristics and associated neurobiological alterations.

In this study, prenatal VPA exposure induced ASD-like behavioral abnormalities in both male and female adolescent offspring, including social deficits, reduced locomotor activity, and anxiety-like behavioral alterations. These behavioral changes were more pronounced in male offspring. Meanwhile, prenatal VPA exposure also triggered neuronal morphological changes in multiple brain regions, such as the mPFC and hippocampus, yet no statistically significant sex difference was observed in these structural alterations. Among all detected brain regions, the mPFC exhibited the most distinct morphological impairments and ferroptosis-related dysregulations. Notably, a prominent sex-related difference was observed specifically in the ferroptosis-related alterations, with more pronounced changes in VPA-exposed male mice than in VPA-exposed female mice.

### Sex differences in behavioral and neuropathological alterations after prenatal VPA exposure

An important finding of this study was that prenatal VPA exposure produced both shared and sex-related behavioral abnormalities. In the OFT, VPA-exposed male and female offspring exhibited reduced locomotor activity and spent less time in the center area, indicating reduced exploratory behavior and a behavioral profile consistent with increased anxiety-like responses in both sexes. However, clear sex-related differences emerged in the 3-CT. Male VPA-exposed offspring retained a tendency to approach social stimuli but exhibited an abnormal pattern of social preference, suggesting that initial social approach may be relatively preserved whereas the motivational or reward-related processes required to assign sustained value to social stimuli may be disrupted [45]. This interpretation is consistent with evidence that the mesolimbic dopaminergic system and its projections to the striatum and nucleus accumbens contribute to the encoding of social reward value [46–48]. Thus, the altered social behavior observed in male VPA-exposed offspring may, at least in part, reflect reduced reward salience of social stimuli. In contrast, female VPA-exposed offspring showed no clear social preference and concurrently displayed reduced exploration and center avoidance in the OFT. These findings raise the possibility that impaired social behavior in females may be accompanied by broader alterations in anxiety-related responses, novelty processing, or risk evaluation [49]. Collectively, these results indicate that prenatal VPA exposure disrupts social behavior in both sexes, but the behavioral expression of this disruption may differ between males and females.

To further explore the neuropathological basis of these behavioral differences, we examined morphological changes in the mPFC, hippocampus, and striatum. The mPFC plays a critical role in social decision-making, risk assessment, and behavioral execution [50, 51]; the hippocampus participates in contextual processing and anxiety regulation [52]; and the striatum is an important node for social reward processing and motivated behavior [53]. HE and Nissl staining showed obvious neuronal damage in the mPFC of both male and female VPA-exposed offspring, including nuclear pyknosis, hyperchromasia, and cell shrinkage. The hippocampal CA3 region showed tissue rarefaction and reduced Nissl-positive neurons, whereas no obvious neuronal morphological damage was observed in the striatum. Notably, despite the sex-related differences in social behavior, the numbers of Nissl-positive neurons in the mPFC, hippocampus, and striatum did not differ significantly between male and female VPA-exposed offspring. This dissociation suggests that the sex-specific behavioral phenotypes are unlikely to be explained simply by differences in the extent of gross neuronal loss. Instead, neuronal injury may represent a largely shared neuropathological consequence of prenatal VPA exposure, whereas sex-related behavioral differences may reflect distinct molecular or functional responses superimposed on this common structural background [54–56]. In this context, alterations in synaptic organization, neuronal excitability, neurotransmitter signaling, and network-level processing may contribute to sex-related differences in behavioral expression without necessarily being accompanied by detectable differences in neuronal number.

The prominent morphological abnormalities observed in the mPFC further suggest that prefrontal injury may constitute an important anatomical substrate of VPA-induced ASD-like behaviors. However, the neurobiological effects of prenatal VPA exposure are unlikely to be restricted to a single brain region. Social behavior depends on coordinated activity across a distributed network involving the mPFC, hippocampus, striatum, and associated mesolimbic structures. Previous studies have shown that prenatal VPA exposure induces brain-region-specific alterations in synaptic organization, synaptic protein expression, oxidative homeostasis, and microglial activity in the cerebral cortex and hippocampus [57, 58]. Schiavi et al. also reported that VPA-exposed animals exhibited impaired sociability and abnormal social preference, accompanied by changes in reward-related behavior, neurochemistry, and electrophysiology [59]. Therefore, the absence of obvious striatal abnormalities in HE and Nissl staining does not exclude striatal dysfunction [60]. Abnormalities in social reward circuits may occur at the level of dopaminergic signaling, receptor expression, synaptic plasticity, or cortico-striatal connectivity rather than overt neuronal loss [59, 61]. Our results indicate that prenatal VPA exposure may induce ASD-like behavioral abnormalities through region- and sex-related disruption of an mPFC-centered social behavior network. However, the relatively mild morphological changes observed in the hippocampus and striatum suggest that structural neuronal loss alone may not fully account for the behavioral phenotypes. Therefore, molecular forms of neuronal dysfunction or regulated cell death should be further considered.

### Sex differences in ferroptosis-related alterations and mPFC vulnerability after prenatal VPA exposure

Ferroptosis is defined by iron-dependent lipid peroxidation and impaired antioxidant defense systems [62]. Accumulating evidence indicates that ferroptosis-related mechanisms may contribute to neurodevelopmental abnormalities and neurological disorders [22, 63, 64]. Neurons may be particularly vulnerable to ferroptosis because of their high metabolic activity, high oxygen consumption, and enrichment of polyunsaturated fatty acids in cellular membranes, which render them susceptible to iron-dependent lipid peroxidation [24]. HE and Nissl staining revealed obvious neuronal damage, providing a histopathological basis for further examining ferroptosis-related alterations. This rationale is also supported by recent evidence showing that ferroptosis contributes to VPA-induced ASD-like phenotypes, including ferroptotic injury of VTA dopaminergic neurons and hippocampal ferroptosis abnormalities in VPA-exposed mice [19, 29]. Our results showed that prenatal VPA exposure triggered a ferroptosis-associated molecular and biochemical profile in the mPFC, hippocampus, and striatum. These alterations included disrupted iron homeostasis, increased Fe²⁺ levels, enhanced ROS-related fluorescence, elevated MDA levels, and upregulated ACSL4 expression, together with reduced GSH levels, decreased SOD activity, and downregulation of GPX4, SLC7A11/xCT, and FTH1. The mitochondrial ultrastructural abnormalities observed by TEM further support ferroptosis as a potential link between oxidative stress, lipid peroxidation, and neuronal dysfunction. Another important finding of this study is that male offspring were more susceptible to ferroptosis-associated changes, particularly in the mPFC. Previous studies suggest that females may generally exhibit stronger antioxidant capacity, lower oxidative stress burden, and greater resistance to lipid peroxidation, partly due to estrogen-mediated regulation of mitochondrial ROS production, antioxidant defense, iron homeostasis, and ferroptosis-related pathways [55, 65, 66]. Estrogen has been reported to enhance antioxidant defenses and maintain mitochondrial function, whereas males may be more vulnerable to oxidative and lipid peroxidation injury under neurodevelopmental stress [67, 68]. Therefore, the more pronounced ferroptosis-related alterations observed in the male mPFC may reflect both greater male susceptibility and potential female protective mechanisms. In this study, male VPA-exposed offspring exhibited more pronounced ferroptosis-related alterations in the mPFC. Together with the behavioral findings and considering the critical role of the mPFC in social decision-making, behavioral flexibility, and top-down regulation of reward- and anxiety-related circuits, these results suggest that more pronounced ferroptosis-associated alterations in the male mPFC may disrupt social stimulus evaluation and reward-guided behavioral processing, thereby contributing to abnormal social preference and reduced social motivation. In contrast, although female VPA-exposed offspring also exhibited ferroptosis-related alterations, the comparatively less pronounced changes in the mPFC may indicate partial protection against VPA-induced ferroptotic stress.

### Ferroptosis-related mPFC alterations across different developmental windows of VPA exposure

The above results indicate that prenatal VPA exposure induces ferroptosis-related molecular injury in a brain-region- and sex-specific manner, with the male mPFC exhibiting particularly pronounced vulnerability. However, whether this heightened susceptibility of the mPFC to ferroptotic stress is unique to prenatal VPA exposure or represents a convergent pathological response to VPA across different developmental windows remains unclear. This question is important because the biological consequences of VPA exposure may depend on the developmental stage at which exposure occurs. Prenatal VPA exposure, particularly around E12.5 in rodents, is widely used to establish ASD-like models and coincides with a critical period of neurogenesis, neuronal migration, and early cortical circuit formation [69–71]. In contrast, early postnatal exposure occurs during a period characterized by synaptogenesis, maturation of excitatory/inhibitory balance, glial development, and refinement of prefrontal cortical circuits [72–74]. Previous studies have shown that ASD-like behavioral abnormalities and neurodevelopmental alterations can be induced both prenatal and postnatal [75, 76]. Moreover, recent evidence showing that PND14 VPA exposure is associated with hippocampal ferroptosis-related abnormalities further supports the possibility that ferroptosis may participate in postnatal VPA-induced ASD-like phenotypes [29]. From this perspective, comparing E12.5 and PND14 VPA exposure provides a useful strategy to distinguish developmental-window-specific effects from shared downstream mechanisms. If both exposure paradigms produce similar ferroptosis-associated alterations in the mPFC, this would suggest that ferroptotic dysregulation is not solely secondary to prenatal disruption of embryonic brain development, but may represent a broader response of the developing brain to VPA-induced stress. Based on this rationale, and given the male-biased ferroptosis vulnerability observed after prenatal VPA exposure, we further focused on male mice to compare the effects of VPA exposure during E12.5 and PND14. In this study, both E12.5 and PND14 VPA exposure induced ASD-like behavioral abnormalities at PND24 and were accompanied by ferroptosis-associated alterations in the male mPFC. Notably, the overall patterns of these ferroptosis-related changes did not differ markedly between the two exposure paradigms. These findings suggest that mPFC ferroptotic dysregulation may not be restricted to prenatal VPA exposure, but may represent a convergent molecular response to VPA-induced neurodevelopmental stress across the prenatal and early postnatal exposure windows.

### Limitations of the study

A major strength of this study is that it integrates sex comparison, brain-region-specific analysis, and developmental-window comparison, thereby providing a more comprehensive view of VPA-induced ferroptosis-related neurodevelopmental abnormalities. However, several limitations should be acknowledged. First, although multiple biochemical, molecular, and ultrastructural indicators were consistent with ferroptosis-associated dysregulation, the present study did not include pharmacological or genetic interventions specifically targeting ferroptosis. Therefore, our findings support an association between ferroptosis-related alterations and VPA-induced neuronal and behavioral abnormalities but do not establish ferroptosis as a causal or exclusive mechanism. Future studies using ferroptosis inhibitors, iron chelators, or genetic manipulation of key ferroptosis regulators will be required to determine whether suppression of ferroptotic processes can ameliorate neuronal injury and ASD-like behavioral phenotypes. Second, this study did not perform cell type-specific analyses. Because ferroptosis-related signals may arise from neurons, astrocytes, microglia, or oligodendrocytes, future studies should determine the cell-type-specific sources of these alterations. Third, behavioral tests were conducted during the light phase under a conventional 12-h light/dark cycle, when mice are generally less active. Although treatment groups and sexes were tested in a randomized and balanced order across the testing period, and tissue collection was performed at a consistent time of day, these procedures minimize systematic timing-related bias but cannot fully exclude an influence of circadian phase on behavioral or molecular outcomes. Previous studies indicate that the effects of testing phase may be paradigm-dependent, with some measures of social approach remaining relatively stable across light and dark phases, whereas other behavioral and molecular readouts may vary with testing phase [77, 78]. In addition, sex hormone levels and sleep–wake state were not directly assessed, and their potential contributions to sex-related variability cannot be excluded [79]. Future studies incorporating dark-phase testing or a reversed light/dark cycle would help determine whether circadian phase modulates the magnitude or expression of VPA-associated behavioral phenotypes. Fourth, the present regional analysis was restricted to the mPFC, hippocampus, and striatum and therefore did not encompass all brain regions relevant to ASD. In particular, the cerebellum was not systematically examined. Previous studies have demonstrated cerebellar abnormalities, particularly those involving Purkinje cells, following developmental VPA exposure [80, 81], while more recent evidence has identified abnormal ferritinophagy, iron accumulation, and lipid peroxidation in cerebellar Purkinje cells of VPA-exposed mice [33]. Therefore, whether ferroptosis-associated alterations also occur in the cerebellum following prenatal and early postnatal VPA exposure, and whether such alterations exhibit sex- or developmental-stage-related patterns, remains to be determined. Fifth, we did not examine individual-level relationships between ferroptosis-related alterations and the severity of behavioral or neuroanatomical abnormalities. Consequently, although ferroptosis-associated dysregulation occurred in parallel with behavioral deficits and neuronal injury, it remains unclear whether animals exhibiting greater disturbances in iron homeostasis, lipid peroxidation, or antioxidant defense also exhibit more severe behavioral or histopathological phenotypes. Future studies integrating behavioral, histological, and molecular measurements at the individual level may help establish quantitative relationships among these alterations, although such correlations alone would not establish causality.

Taken together, these limitations indicate that the present findings identify a close association between VPA exposure, ferroptosis-associated dysregulation, neuronal injury, and ASD-like behavioral abnormalities, while the cellular origin, phenotype–molecular relationships, circadian influences, and causal contribution of ferroptosis remain to be fully defined. Further studies combining ferroptosis-targeted interventions with cell type-resolved analyses and temporally controlled behavioral and molecular assessments will be required to clarify the mechanistic role of ferroptosis in VPA-induced neurodevelopmental abnormalities.

## Conclusion

In summary, prenatal VPA exposure at E12.5 induced ASD-like behavioral abnormalities and neuronal injury in both male and female offspring, while the behavioral phenotypes and ferroptosis-associated alterations exhibited sex-related differences. In particular, male offspring showed greater susceptibility to ferroptosis-associated dysregulation. Among the examined brain regions, including the mPFC, hippocampus, and striatum, the mPFC exhibited prominent morphological abnormalities together with the most pronounced ferroptosis-associated changes, identifying it as a potentially vulnerable region in VPA-induced neurodevelopmental pathology. Notably, ferroptosis-associated alterations in the male mPFC were observed following VPA exposure at both E12.5 and PND14, suggesting that such dysregulation may represent a convergent molecular response across the prenatal and early postnatal exposure windows examined in this study. Collectively, these findings suggest that ferroptosis-associated dysregulation may represent an important molecular component of VPA-induced neuronal injury and ASD-like behavioral abnormalities, particularly in the male mPFC, although its causal contribution remains to be established.

## Electronic Supplementary Material

Below is the link to the electronic supplementary material.

Supplementary Material 1

## Data Availability

No datasets were generated or analysed during the current study.

## Abbreviations

ASD: Autism spectrum disorder
VPA: Valproic acid
3-CT: Three-chamber test
OFT: Open field test
Hpc: Hippocampus
mPFC: Medial prefrontal cortex
Sti: Striatum
TEM: Transmission electron microscopy
WB: Western blotting
Gpx4: Glutathione peroxidase 4
ROS: Reactive Oxygen Species
SOD: Superoxide Dismutase
MDA: Malondialdehyde
GSH: Glutathione
PBS: Phosphate-buffered solution
BSA: Bovine serum albumin
CA1: Cornu ammonis 1
CA3: Cornu ammonis 3
DG: Dentate gyrus
ACSL4: Acyl-CoA synthetase long-chain family member 4
FTH1: Ferritin heavy chain 1
