# Supplementary material for: Sex differences in ferroptosis-related vulnerability to autism-like deficits in the adolescent medial prefrontal cortex following embryonic valproic acid exposure
Source: Biol Sex Differ. 2026 Sep 15;17:152. doi: 10.1186/s13293-026-00982-x (PMC13579844; doi:10.1186/s13293-026-00982-x)
Supplement: Supplementary file 1 [file 13293_2026_982_MOESM1_ESM.zip › Supplementary_Figure.docx]

**Supplementary Figure. S1 Developmental characteristics of offspring following VPA exposure. A-D** Comparison of body weight, body length, tail length and brain wet weight in VPA-induced mice at E12.5. **E-H** Comparison of differences in body weight, body length, tail length and brain wet weight between prenatal and postnatal VPA-induced mice. *represent saline male vs vpa male, ES vs EV. **P* < 0.05, ***P* < 0.01, and ****P* < 0.001. #represents saline female vs vpa female, PS vs PV. ^#^*P* < 0.05, ^##^*P* < 0.01. One-way ANOVA was used to assess group main effects and interactions. Data are presented as mean ± SEM. ES: E12.5 Saline-exposed. PS: PND14 Saline-exposed. EV: E12.5 VPA-exposed. PV: PND14 VPA-exposed.
